# Unbalanced selection: the challenge of maintaining a social polymorphism when a supergene is selfish

**DOI:** 10.1098/rstb.2021.0197

**Published:** 2022-08-01

**Authors:** Alireza G. Tafreshi, Sarah P. Otto, Michel Chapuisat

**Affiliations:** ^1^ Department of Zoology and Biodiversity Research Centre, University of British Columbia, Vancouver, British Columbia, Canada V6T 1Z4; ^2^ Department of Ecology and Evolution, University of Lausanne, 1015 Lausanne, Switzerland

**Keywords:** genetic polymorphism, heterozygote advantage, selfish genetic element, social organization, ants, invasion analysis

## Abstract

Supergenes often have multiple phenotypic effects, including unexpected detrimental ones, because recombination suppression maintains associations among co-adapted alleles but also allows the accumulation of recessive deleterious mutations and selfish genetic elements. Yet, supergenes often persist over long evolutionary periods. How are such polymorphisms maintained in the face of selection, drive and drift? We present a population genetic model that investigates the conditions necessary for a stable polymorphic equilibrium when one of the supergene haplotypes is a selfish genetic element. The model fits the characteristics of the Alpine silver ant, *Formica selysi*, in which a large supergene underlies colony social organization, and one haplotype distorts Mendelian transmission by killing progeny that did not inherit it. The model shows that such maternal-effect killing strongly limits the maintenance of social polymorphism. Under random mating, transmission ratio distortion prevents rare single-queen colonies from invading populations of multiple-queen colonies, regardless of the fitness of each genotype. A stable polymorphic equilibrium can, however, be reached when high rates of assortative mating are combined with large fitness differences among supergene genotypes. The model reveals that the persistence of the social polymorphism is non-trivial and expected to occur only under restrictive conditions that deserve further empirical investigation.

This article is part of the theme issue ‘Genomic architecture of supergenes: causes and evolutionary consequences’.

## 1. Introduction

Supergenes—large non-recombining genomic regions—underlie some of the more striking polymorphisms in nature [1,2]. They commonly affect multiple traits of the phenotype, controlled by linked alleles, of which some are co-adapted [3,4]. Supergenes are also prone to accumulate recessive deleterious mutations [2,5] and selfish genetic elements that distort the laws of Mendelian inheritance [6–8]. Indeed, the lack of recombination hinders the purging of deleterious elements and allows the accumulation of selfish genetic elements that favour their own transmission, such as toxin-antidote elements [9]. Yet, supergenes often persist over long evolutionary periods [10]. How are such polymorphisms balanced in the face of selection, drive and drift? Fundamental mechanisms leading to balanced polymorphisms at supergenes include various forms of negative frequency-dependent selection, temporally or spatially varying selection, overdominance and associative overdominance [2,5,11]. Because supergenes have complex effects, understanding their evolutionary trajectory remains a substantial challenge.

A supergene usually arises when recombination is suppressed, often as a result of one or more inversions or other structural changes [12], so that a group of neighbouring genes becomes inherited as a single Mendelian element. The long-term fate of the novel haplotype will depend on drift and on the combined selective effects over multiple supergene elements. A mutant non-recombining haplotype will spread if it has captured adaptive combinations of alleles (the supergene hypothesis; reviewed in [4]). Non-recombining haplotypes can also spread selfishly, by being transmitted to a disproportionate number of adult offspring. Indeed, gene drive arises when a driver locus (typically with toxic ‘killer’ effects on the product of a responder locus) becomes tightly linked to an insensitive allele at the responder locus, so that the selfish genetic element is not suicidal [6,9]. Such selfish genetic elements tend to be located in non-recombining regions, like supergenes [8,13]. Counteracting these advantages, non-recombining haplotypes are predicted to accumulate deleterious mutations [5]. Hence, in many cases, homozygous lethality prevents a positively selected or driven haplotype from reaching fixation [1,2]. As supergenes influence multiple traits at once, which often have confounding or antagonistic effects, formal modelling is needed to understand their evolutionary dynamics.

**Figure 1.**
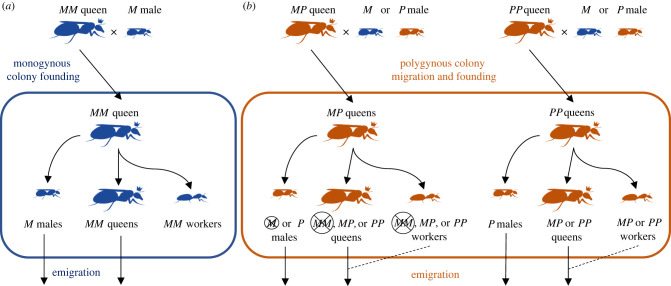
Social and genetic system of *F. selysi.* (*a*) Mature monogynous colonies contain a single *MM* queen mated with *M* males. The queen produces *M* males (haploid, from unfertilized eggs), as well as *MM* queens and *MM* workers (diploid, from fertilized eggs). The offspring (males and queens) fly out of the colony for mating, and queens establish colonies independently. (*b*) Mature polygynous colonies contain multiple *MP* or *PP* queens mated with *M* or *P* males. The offspring (queens and males) also fly out of the colony for mating. *MP* and *PP* queens (and possibly *MM* queens mated to *P* males) may establish colonies independently, or, for polygynous queens, with the help of workers from their natal colony (dashed line). Th*e P* haplotype acts as a maternal-effect killer, so that all offspring of *MP* queens that do not inherit the *P* haplotype die during development. As a result, *M* males and *MM* females are never produced by polygynous colonies. (Online version in colour.)

Here, we present a model examining the conditions for the long-term persistence of a polymorphism in a supergene controlling alternative forms of social organization in ants (reviewed in [14]), in which one haplotype is a transmission ratio distorter. The model is designed to fit the properties of the Alpine silver ant, *Formica selysi*. This species is polymorphic for colony social organization: within the same populations, it forms monogynous colonies, which have a single reproductive queen, and polygynous colonies, in which multiple queens share offspring production [15–17]. A genome-wide association study coupled with linkage maps has revealed that a large supergene with two highly differentiated non-recombining haplotypes, *Sm* and *Sp*, underlies this social polymorphism [18]. We will simplify the notation slightly and refer to the *Sm* haplotype as *M* and the *Sp* haplotype as *P*. Mature (=large-sized, several years old) monogynous colonies consist of individuals carrying exclusively the *M* haplotype: all females (queens and workers) have the supergene genotype *MM*, while all males have the haplotype *M* (females are diploid and males haploid in ants; figure 1*a*). In contrast, polygynous colonies consist of female ants carrying at least one copy of the *P* haplotype, i.e. having the supergene genotype *PP* or *MP*, and producing only *P* males (figure 1*b*). Monogynous colonies are established by *MM* queens independently, without the help of workers, whereas polygynous colonies are founded by *MP* or *PP* queens, and possibly *MM* queens mated to *P* males, either independently or accompanied by workers from their natal colony (figure 1).

The supergene controlling social organization in *F. selysi* is very ancient, being shared by multiple *Formica* lineages separated by 20–40 Myr of independent evolution [19]. The two haplotypes have accumulated substantial sequence divergence and differ by several inversions [18,19]. Field data on *F. selysi* suggest that the proportion of monogynous and polygynous colonies tends to be stable across years, remaining close to 50% in one well-studied population [15,17], while varying between patches of mosaic habitat within populations [20]. Moreover, many populations contain both types of colonies [21]. Overall, the comparative genomic and population genetic data show the signature of strong and persistent balancing selection, consistent with an evolutionarily stable polymorphism.

Genetic and behavioural data indicate ongoing gene flow between the monogynous and polygynous social forms of *F. selysi*. First, the two social forms show no or minimal signs of genetic differentiation across most of their genome, outside of the supergene [18,22]. Second, monogynous and polygynous colonies are found in close spatial proximity within the same populations [15,20,21]. Third, virgin queens and males fly to mating sites on neighbouring trees, where they mate [15,23]. Queens and males from alternative social forms readily mate in choice experiments [24], as well as in field mating swarms [23]. In mature field colonies, the observed pattern of non-random mating suggests unidirectional gene flow from the monogynous to the polygynous social form ([22]; figure 1). Moreover, recent analyses of mating swarms and incipient colonies revealed that *MM* females do mate with *P* males [23,25]. These queens might establish colonies that do not reach a mature size or that become polygynous, given that in the field, we never detected a mature colony headed by a single monogynous queen mated with a *P* male [18,22].

Mating in Alpine silver ants is non-random with respect to the social form of queens and males [18,22,23]. The sampling and genotyping of ants from mature field colonies revealed that queens from monogynous colonies had mated exclusively with *M* males (*n* = 69 matings), while queens from polygynous colonies had mated with both *P* and *M* males, the latter accounting for 23% of the matings, for both *MP* and *PP* queens (*n* = 190 matings) [22]. While matings outside of the colony have been well documented [15,23], evidence for within-colony mating is only indirect [22]. In monogynous colonies, mates were not significantly related to queens, suggesting that within-colony mating is rare or absent in this social form. In polygynous colonies, mates were slightly related to queens, suggesting that some of the queens had mated with nest-mate males within or near the natal colony [22]. Queens occasionally mate with multiple males [15,22]. Local mate availability or mate preferences, as well as sperm discrimination or fertility differences after mating, may influence the probability of each cross. After mating, both types of queens are able to establish colonies independently [25]. In addition, polygynous queens may establish colonies with workers from their natal colony (colony budding). Overall, due to pronounced differences in life-history, including dispersal, mating and colony founding [16,23], queens from alternative social forms are likely to greatly differ in fitness.

A striking feature of the genetic system underlying this social polymorphism is that the *P* haplotype acts as a maternal-effect killer, causing the early death of any offspring of *MP* mothers that do not bear the *P* haplotype ([8]; figure 1*b*). While *MP* females commonly mate with *M* males and produce *MP* and *MM* eggs in Mendelian proportions (no meiotic drive), *MM* or *M* eggs from *MP* mothers fail to hatch [8,22]. Thus, *MP* females never produce adult *MM* daughters or *M* sons (figure 1*b*). In short, the *P* haplotype causes complete gene drive, distorting Mendelian transmission in such a way that all offspring produced by polygynous colonies carry a *P* haplotype. Furthermore, substantial fitness differences have been detected among females carrying alternative genotypes, in both the laboratory and the field (P Blacher, O De Gasperin, G Grasso, S Sarton-Lohéac, R Allemann and M Chapuisat 2022, unpublished results).

In this article, we show that the transmission ratio distortion induced by maternal-effect killing strongly limits the maintenance of the social polymorphism. We develop a population genetic model that incorporates the known aspects of the genetic, social and mating systems of *F*. *selysi*. Using this model, we determine the conditions necessary to reach a stable polymorphic equilibrium. Our model reveals that, under many conditions, classical forms of balancing selection—including when selfish genetic elements are balanced by strong counterselection in homozygotes—fail to stabilize the social polymorphism. In other examples of transmission ratio distortion (e.g. tailless in mice), an element that is driven (e.g. the *t* allele) spreads when rare but is prevented from fixing by the sterility of *tt* males [26]. With *F*. *selysi*, however, maternal-effect killing prevents *MP* females from producing the *MM* females that are needed to establish monogynous colonies. Thus, while low fitness of the driven haplotype *P* can ensure that *M* spreads when rare, the result is generally an equilibrium consisting of only *PP* and *MP* females in polygynous colonies, and the social polymorphism is lost. Even if maternal-effect killing is not complete and the occasional *M* son or *MM* daughter is produced, they would generally fail to establish new monogynous colonies, because most of their matings would be with polygynous individuals, whose offspring are of the polygynous type. Hence, when mating is random, maternal-effect killing leads to the extinction of monogynous colonies regardless of how strong selection is against homozygotes. Overall, as we will show, the gene drive caused by maternal-effect killing destabilizes the genetic polymorphism and precludes the maintenance of a social polymorphism with both polygynous and monogynous colonies when mating is random and fitnesses are frequency-independent. The goal of this paper is to determine what exactly could account for the maintenance of the supergene polymorphism and both social forms.

Here, we briefly outline the approach that we take, section by section. We start by constructing a general model that can be used to explore all scenarios described below. We then apply this model to different forms of reproduction, starting with random mating, to determine the conditions under which the social polymorphism observed in *F*. *selysi* would be stable:

(1) Random mating: we first considered the case of a randomly mating population, allowing for arbitrarily strong natural selection, finding that the social polymorphism is never stable.
(2) Variation in fertility: we then include fertility differences between mating pairs, again finding that the social polymorphism is never stable.
(3) Sexual selection: next, we incorporate sexual selection, using the fixed-relative preference scheme of Kirkpatrick [27]. This model assumes that all queens will be mated and that their preferences determine the relative frequency of mating with *M* and *P* males. Again, we show that a social polymorphism is not maintained except when sexual selection is so extreme that it results in assortative mating.
(4) Assortative mating: finally, we model assortative mating, finding that partial (but not complete) assortative mating can maintain the social polymorphism if combined with strong enough selection.

Together, these models demonstrate how challenging it is to maintain the *F. selysi* social polymorphism because of the selfish supergene drive of the *P* haplotype and clarify the conditions required to account for the persistence of both monogynous and polygynous colonies.

## 2. Model

Motivated by *F. selysi*, we develop a population genetic model to investigate the conditions necessary for a stable polymorphic equilibrium when one haplotype is a maternal-effect killer. This model with non-overlapping generations and male haploidy follows the dynamics of genotypes at a supergene controlling social organization among reproductive females (=queens) and among males separately (table 1). Because workers do not reproduce (figure 1), we do not incorporate them within the population dynamics, except indirectly via the fitness of queens and males. We census at the adult stage, among surviving ants, who then mate and produce offspring that are subjected to selection (see electronic supplementary material, appendix table for definitions of all parameters).

**Table 1.** Genotype frequencies among adult queens and males. Social form refers to the social organization of the colony of origin.

supergene genotype	sex	social form	frequency
*MM*	queen	monogynous	$X_{MM}$
*MP*	queen	polygynous	$X_{MP}$
*PP*	queen	polygynous	$X_{PP}$
*M*	male	monogynous	$Y_{M}$
*P*	male	polygynous	$Y_{P}$

To model the production of males, which is not dependent on mating, we let $O_{a|ij}^{\mathrm{male}}$ represent the production rate of surviving male offspring of genotype *a* produced by a queen of genotype *ij*. We assume Mendelian segregation but allow the number and survival of the male offspring, $V_{a|ij}^{\mathrm{male}}$, to depend on its mother's genotype, enabling us to incorporate maternal effects, including maternal-effect killing, and effects of colony type on fitness. For example, *MP* queens at frequency *X_MP_* produce *M* and *P* males at rates proportional to

2.1*a* $$O_{M|MP}^{\mathrm{male}}=\frac{1}{2}X_{MP}\;V_{M|MP}^{\mathrm{male}}$$

and

2.1*b* $$O_{P|MP}^{\mathrm{male}}=\frac{1}{2}X_{MP}\;V_{P|MP}^{\mathrm{male}}.$$

If there is complete maternal-effect killing of all offspring that did not inherit the *P* haplotype from their *MP* mother, then $V_{M|MP}^{\mathrm{male}}=0$. If colonies are unable to recover the resources invested in lost males, $V_{P|MP}^{\mathrm{male}}$ is expected to be near one (all else being equal). If colonies are able to fully recover the energy invested in lost embryos and use it to produce *P* males, then $V_{P|MP}^{\mathrm{male}}$ may be elevated relative to other male fitnesses.

To model the production of queens, we let $O_{ab|ij\times k}^{\mathrm{female}}$ represent the production rate of surviving queen offspring of genotype *ab* produced from matings between a queen of genotype *ij* and a male of genotype *k*. As described below, $O_{ab|ij\times k}^{\mathrm{female}}$ incorporates both the reproductive mode and several components of fitness, including the rate at which different crosses occur, fertility differences, offspring number, and the survival rate of daughters, $V_{ab|ij\times k}^{\mathrm{female}}$ (table 2). We assume that the zygotes are produced in a Mendelian fashion but allow complete maternal-effect killing, eliminating *MM* offspring of *MP* mothers $\left(V_{MM|MP\times M}^{\mathrm{female}}=0\right)$.

**Table 2.** Production of queens.

mating	frequency	viability of *ab* offspring	proportion of *MM* offspring	proportion of *MP* offspring	proportion of *PP* offspring
*MM × M*	$R_{MM\times M}$	$V_{ab|MM\times M}^{\mathrm{female}}$	1	0	0
*MM × P*	$R_{MM\times P}$	$V_{ab|MM\times P}^{\mathrm{female}}$	0	1	0
*MP × M*	$R_{MP\times M}$	$V_{ab|MP\times M}^{\mathrm{female}}$	1/2	1/2	0
*MP × P*	$R_{MP\times P}$	$V_{ab|MP\times P}^{\mathrm{female}}$	0	1/2	1/2
*PP × M*	$R_{PP\times M}$	$V_{ab|PP\times M}^{\mathrm{female}}$	0	1	0
*PP × P*	$R_{PP\times P}$	$V_{ab|PP\times P}^{\mathrm{female}}$	0	0	1

To model mating, we introduce an arbitrary reproductive function, $R_{ij\times k}$, that describes the frequency of crosses between queens of genotype *ij* and males of genotype *k*. This function depends on the reproductive mode considered in each section of the results:

(1) Random mating: when mating is random, *ij* × *k* crosses occur at a frequency of $R_{ij\times k}=X_{ij}\;Y_{k}$.
(2) Variation in fertility: if there are fertility differences among mating pairs $\left(f_{ij\times k}\right)$, but mating is otherwise random, we set $R_{ij\times k}=f_{ij\times k}X_{ij}\;Y_{k}$, where $f_{ij\times k}$ is measured relative to the mean fertility in each generation.
(3) Sexual selection: using the fixed-relative preference scheme of Kirkpatrick [27], females of genotype *ij* mate with each type of male *k* in proportion to their mating preference $\left(\alpha_{ij\times k}\right)$, leading to $R_{ij\times k}=X_{ij}(\alpha_{ij\times k}Y_{k}/\left(\alpha_{ij\times P}Y_{P}+\alpha_{ij\times M}Y_{M}\right))$, where females are assumed to be able to mate with another male after rejecting a candidate. If mating preferences are costly, this is assumed to be incorporated into the female's viability $\left(V_{ab|ij\times k}^{\mathrm{female}}\right)$, regardless of the composition of the male population (a consititutive cost of sexual selection).
(4) Assortative mating: finally, we consider assortative mating by social type with $R_{MM\times M}=(1-m_{M})$ $X_{MM}\;Y_{M}+m_{M}\;X_{MM}$, where a proportion $m_{M}$ of all *MM* queens mate with males from their own social form, regardless of the frequency of *M* males (see equation (3.6) for additional crosses).

Importantly, except with assortative mating, the frequency of matings involving rare genotypes is proportional to the frequency of the rare female times the frequency of the rare male (e.g. proportional to $X_{MM}\;Y_{M}$ when both *MM* females and *M* males are rare).

Overall, the production of *MP* queens from matings between *MP* queens and *P* males, for example, is proportional to

2.2 $$O_{MP|MP\times P}^{\mathrm{female}}=\frac{1}{2}R_{MP\times P}\;V_{MP|MP\times P}^{\mathrm{female}}.$$

Finally, we normalize the frequencies in each sex to obtain the genotype frequencies in the next generation among queens and males, respectively

2.3*a* $$X_{ab}^{{\;}^{\prime }}=\frac{\sum_{ij,k}O_{ab|ij\times k}^{\mathrm{female}}}{\sum_{ab,ij,k}O_{ab|ij\times k}^{\mathrm{female}}}$$

and

2.3*b* $$Y_{a}^{{\;}^{\prime }}=\frac{\sum_{ij}O_{a|ij}^{\mathrm{male}}}{\sum_{a,ij}O_{a|ij}^{\mathrm{male}}},$$

where the sums in the numerator are taken over all maternal (*ij*) and, for female offspring, paternal genotypes (*k*), and the sums in the denominator are also taken over all offspring genotypes (*ab* for female and *a* for male offspring). Given this normalization, all fitnesses need only be measured relative to one another within a sex, so we consider $V_{a|ij}^{\mathrm{male}}$ and $V_{ab|ij\times k}^{\mathrm{female}}$ to vary between 0 and 1 in our numerical analysis.

*F. selysi* queens may differ in how they form new colonies. Queens from monogynous colonies fly away from their natal colony and establish novel colonies independently, while queens from polygynous colonies may also establish novel colonies with the help of workers, by walking away from their natal colony (colony budding; [15,28]). Furthermore, polygynous colonies are larger than monogynous colonies, in line with their greater number of queens and longer colony lifespan [16]. Queens of monogynous origin may, however, be more successful at establishing new colonies independently, compared to queens of polygynous origin [24,29]. We do not explicitly model colony dynamics but include any fitness differences in colony success through the fitness terms, $V_{a|ij}^{\mathrm{male}}$ and $V_{ab|ij\times k}^{\mathrm{female}}$, which also allows for the possibility that a queen's fitness depends on her parents' genotypes through their effect on the colony type and the genotypes of siblings. All of the recursion equations and the full analyses are detailed in the electronic supplementary material, *Mathematica* file.

## 3. Maintenance of the social polymorphism

Below, we analytically search for conditions under which both monogynous and polygynous colonies will increase when rare, indicating that there is a protected polymorphism. We assume that maternal-effect killing is complete [8], with $V_{MM|MP\times M}^{\mathrm{female}}=V_{M|MP}^{\mathrm{male}}=0$, although we discuss the effect of allowing some *M* male and *MM* female offspring to be produced by *MP* mothers. We supplement this analysis with numerical searches, drawing parameters at random (as specified below) and determining numerically all equilibria and their stability properties. These numerical searches were used to confirm the analytical results and to determine whether stable internal polymorphisms could occur even when the polymorphism was not protected (i.e. when monogynous and polygynous colonies could not both increase when rare).

### (a) Random mating

We start by considering the case of random mating, $R_{ij\times k}=X_{ij}\;Y_{k}$. With complete maternal-effect killing, there are three ways that the social polymorphism could be lost from the system at equilibrium: (i) *MM* queens and *M* males are fixed, (ii) *PP* queens and *P* males are fixed, and (iii) *PP* and *MP* queens coexist alongside *P* males. The first of these equilibria consists only of monogynous colonies, while the other two consist only of polygynous colonies, falling along the polygynous edge defined by the absence of *MM* queens and *M* males ($X_{MM}=Y_{M}=0$; recall that maternal-effect killing prevents monogynous genotypes from being produced by the *MP* queens).

A local stability analysis (electronic supplementary material) shows that, under random mating, monogynous fixation with *MM* queens and *M* males is unstable to the introduction of the *P* haplotype when

3.1 $$V_{MM|MM\times M}^{\mathrm{female}}V_{M|MM}^{\mathrm{male}}<\;\frac{V_{MP|MM\times P}^{\mathrm{female}}V_{P|MP}^{\mathrm{male}}+V_{MP|MP\times M}^{\mathrm{female}}V_{M|MM}^{\mathrm{male}}}{2}.$$

This result shows that polygynous colonies can spread when rare if the *P* haplotype confers a higher average fitness, calculated as the average fitness of offspring queens that bear the rare *P* haplotype when the *P* was inherited from the father (first term in the fraction) or the mother (second term), in each case multiplied by the fitness of the father.

Along the polygynous edge, the system equilibrates at either the fixation of *PP* queens and *P* males or an equilibrium with a mixture of *MP* and *PP* queens and *P* males, where the equilibrium frequency of *MP* queens is

3.2 $${\hat{X}}_{MP}=\;\frac{V_{MP|MP\times P}^{\mathrm{female}}-2\;V_{PP|PP\times P}^{\mathrm{female}}}{V_{MP|MP\times P}^{\mathrm{female}}+V_{PP|MP\times P}^{\mathrm{female}}-2\;V_{PP|PP\times P}^{\mathrm{female}}}.$$

Of the two equilibria along the polygynous edge (${\hat{X}}_{MP}$ equal to 0 or to (3.2)), only one is ever stable. Specifically, polygynous colonies consisting of only *PP* queens are unstable to the introduction of *MP* queens if

3.3 $$V_{PP|PP\times P}^{\mathrm{female}}<\frac{V_{MP|MP\times P}^{\mathrm{female}}}{2},$$

in which case, the equilibrium with only *MP* and *PP* queens (3.2) is stable. Conversely, if condition (3.3) fails to hold, then the only stable equilibrium along the polygynous edge is the *PP*/*P* fixation point. Note that maintaining both *MP* and *PP* queens in polygynous-only colonies requires very strong heterozygous advantage, with *MP* queens more than twice as fit as *PP* queens (i.e. equilibrium (3.2) is valid and stable only if $\left(V_{MP|MP\times P}^{\mathrm{female}}>2\;V_{PP|PP\times P}^{\mathrm{female}}\right)$). This requirement for strong selection emerges because, when mated with *P* males, only half of the daughters of *MP* queens are *MP*, whereas all daughters of *PP* queens are *PP*.

We next considered the stability of these polygynous equilibria to the introduction of monogynous genotypes (*MM* queens and *M* males), under random mating. We find that *MM* queens and *M* males never spread when rare, from either the *P*-fixed or *MP*/*PP* equilibria. This is because almost all matings involving a rare *M* male are with the common *PP* or *MP* females, which do not produce *MM* daughters because of maternal-effect killing. Similarly, the vast majority of matings involving the rare *MM* queen are with the common *P* male, which also do not generate *MM* daughters. Thus, the *MM* genotype rapidly disappears from the population of queens, taking along with it the ability to produce *M* males. Even if maternal-effect killing is strong but not complete, such that both $V_{MM|MP\times M}^{\mathrm{female}}$ and $V_{M|MP}^{\mathrm{male}}$ are small but not zero, the monogynous genotypes do not invade under biologically reasonable conditions (with exceptions only in the extreme case where the fitness of *M* males is so much higher than *P* males that rare *M* males become common following a single bout of selection; see electronic supplementary material). This result, that monogynous colonies cannot establish when rare, holds whether or not colonies with *MP* queens are able to recover the energy invested in lost embryos (i.e. whether or not $V_{P|MP}^{\mathrm{male}}$ is elevated due to recovered resources following maternal-effect killing).

In short, if *MP* queens are much fitter than *PP* queens (condition (3.3) holds), then a genetic polymorphism with both *PP* and *MP* queens can result, but the system consists of only polygynous colonies. In particular, no matter how strong heterozygous advantage might be, a social polymorphism cannot be maintained. Polygynous colonies can spread when rare if condition (3.1) holds, and the system converges to one of the polygynous equilibria (with *P*-fixed or with *MP*/*PP* queens), whereas monogynous colonies cannot generally spread when rare. This result is in stark contrast to other systems, where a polymorphism can be stably maintained by heterozygote advantage [30] or a balance between meiotic drive and homozygous sterility/lethality [26].

**Figure 2.**
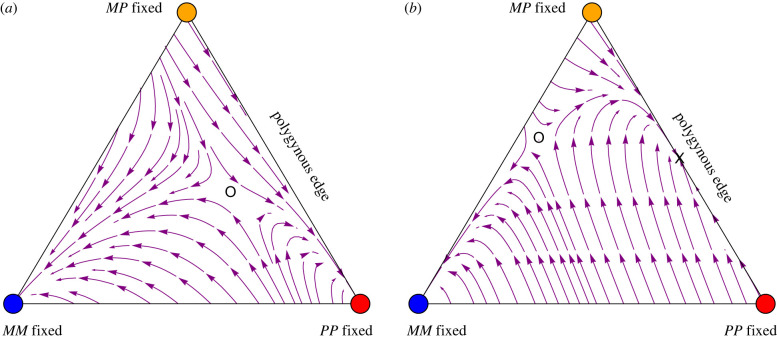
Stream plots show the dynamics of *F. selysi* queens when individuals in monogynous colonies are most fit. The fixation of monogynous colonies (blue) is then stable to the spread of the *P* haplotype (condition (3.1) does not hold because *MM* queens and *M* males are sufficiently fit). (*a*) When heterozygous females are low enough in fitness, the system evolves towards the fixation of either *MM* females (blue) or *PP* females (red) $\left(V_{MP|ij\times k}^{\mathrm{female}}=1/5\right)$. (*b*) When heterozygous females are intermediate in fitness, the system can evolve towards the fixation of either *MM* females (blue) or a polymorphism with both *MP* and *PP* polygynous queens (marked by an ×) ($V_{MP|ij\times k}^{\mathrm{female}}=3/5$). The open circle marks an unstable equilibrium point. Other parameters: $V_{MM|MP\times M}^{\mathrm{female}}=V_{M|MP}^{\mathrm{male}}=0$ (complete maternal-effect killing), $V_{MM|MM\times M}^{\mathrm{female}}=1$ and otherwise $V_{a|ij}^{\mathrm{male}}=V_{ab|ij\times k}^{\mathrm{female}}=1/5$. (Online version in colour.)

**Figure 3.**
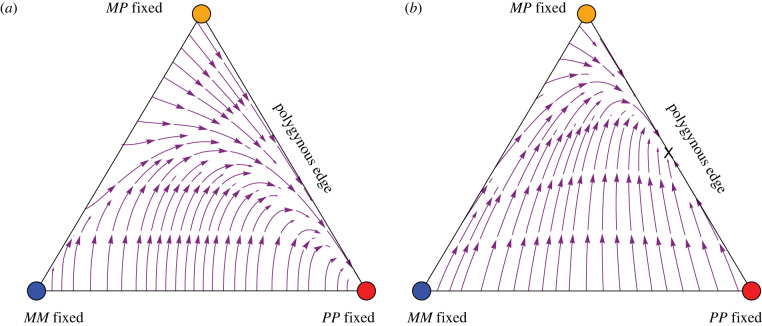
Stream plots show the dynamics of *F. selysi* queens when individuals in monogynous colonies are not more fit than individuals in polygynous colonies. The fixation of monogynous colonies (blue) is then unstable (condition (3.1) holds). (*a*) There are no fitness differences except those caused by maternal-effect killing, in which case *PP*-fixed is the only stable equilibrium (red). (*b*) There is heterozygous advantage, such that condition (3.3) holds, and *MP*/*PP* with only polygynous colonies is the only stable equilibrium (marked by an × on plot; $V_{MP|ij\times k}^{\mathrm{female}}=3/5$). No internal equilibrium point exists in these cases. Other parameters: $V_{MM|MP\times M}^{\mathrm{female}}=V_{M|MP}^{\mathrm{male}}=0$ (complete maternal-effect killing) and otherwise $V_{a|ij}^{\mathrm{male}}=V_{ab|ij\times k}^{\mathrm{female}}=1/5$. (Online version in colour.)

We next illustrate the dynamics of the *F. selysi* system numerically. Because the frequency of males is determined entirely by the genotype frequencies in their mothers (equation (2.3*b*)), we initialize the male frequencies according to equation (2.3*b*) using the current frequency of females, allowing us to closely approximate the dynamics using only genotype frequencies among queens. We then visualize these dynamics using ternary stream plots (figures 2 and 3), where each corner of the triangle represents the fixation of a queen genotype, the opposite edge corresponds to the absence of that genotype, and the distance from the edge to the corner represents the frequency of that queen genotype. Arrows on the stream plot represent the direction of the change in queen frequencies from different initial positions. Figure 2 illustrates a case where the *M*-fixed equilibrium is stable along with either the *P*-fixed equilibrium (figure 2*a*) or the *MP*/*PP* equilibrium (3.2) on the polygynous edge (figure 2*b*). In such cases, a polymorphic internal equilibrium with all social types can exist but is unstable. Figure 3 illustrates a case where the *M*-fixed equilibrium is unstable, in which case the only stable equilibrium is on the polygynous edge, either the *P*-fixed equilibrium (figure 3*a*) or the *MP*/*PP* equilibrium (3.2) (figure 3*b*), and there is no equilibrium with both monogynous and polygynous colonies.

Finally, we verified the above results numerically, confirming that a social polymorphism was never stable using a numerical search. To conduct this search, we randomly drew one million sets of parameters (all chosen uniformly between 0 and 1, other than the maternal-effect killing parameters maintained at $V_{MM|MP\times M}^{\mathrm{female}}=V_{M|MP}^{\mathrm{male}}=0$). For each parameter set, we numerically calculated all equilibria and determined their stability properties, confirming that there was never a stable internal equilibrium under random mating with both monogynous and polygynous colonies (see electronic supplementary material for all numerical results).

### (b) Variation in fertility

Adding fertility differences alters equations (3.1)–(3.3) but does not qualitatively affect the outcome. In particular, there is never a case where both monogynous and polygynous haplotypes can spread when rare, except for biologically extreme male fitness differences. For example, with fertility differences $\left(R_{ij\times k}=f_{ij\times k}\;X_{ij}\;Y_{k}\right)$, condition (3.1) determining when a monogynous social system is unstable to the introduction of polygyny becomes:

3.4 $$\begin{array}{rl} & V_{MM|MM\times M}^{\mathrm{female}}\;f_{MM\times M}\;V_{M|MM}^{\mathrm{male}}\\ & \;<\;\frac{V_{MP|MM\times P}^{\mathrm{female}}\;f_{MM\times P}\;V_{P|MP}^{\mathrm{male}}+V_{MP|MP\times M}^{\mathrm{female}}\;f_{MP\times M}\;V_{M|MM}^{\mathrm{male}}}{2}. \end{array}$$

Similarly, condition (3.3) determining when polygynous-only colonies will evolve towards an equilibrium with both *MP*/*PP* becomes:

3.5 $$V_{PP|PP\times P}^{\mathrm{female}}\;f_{PP\times P}<\frac{V_{MP|MP\times P}^{\mathrm{female}}\;f_{MP\times P}}{2}.$$

Again, however, monogynous colonies can never spread when rare within a system consisting only of polygynous colonies. Furthermore, a numerical search across one million randomly drawn parameter sets failed to find any case of a stable social polymorphism.

Therefore, we have shown that, under a broad range of conditions including arbitrary fertility differences, there is not a protected polymorphism where both monogynous and polygynous forms can spread when rare. Consequently, we would not expect a social polymorphism to be maintained under random mating, with or without fertility differences, because of the strong constraints imposed by maternal-effect killing. Graphically, the problem is that the polygynous edge, consisting of only *PP* and *MP* queens in figures 2 and 3, is always strongly absorbing because neither rare *M* males nor rare *MM* queens produce *MM* daughters: rare *M* males mate predominantly with common *MP*/*PP* queens whose daughters are never *MM* because of maternal-effect killing, and *MM* queens mate predominantly with common *P* males whose daughters are never *MM* by the rules of inheritance.

### (c) Sexual selection

The same conclusions regarding invasion of rare *M* and *P* haplotypes hold true with sexual selection using the fixed-relative preference scheme of Kirkpatrick [27], where $R_{ij\times k}=X_{ij}\;(\alpha_{ij\times k}Y_{k}/\left(\alpha_{ij\times P}\;Y_{P}+\alpha_{ij\times M}\;Y_{M}\right))$. In this case, invasion analysis gives the same result as (3.4), setting $f_{MM\times M}=f_{MP\times M}=1$ and $f_{MM\times P}=\alpha_{MM\times P}/\alpha_{MM\times M}$ (because *P* males are rare and the $Y_{P}$ term in the denominator of $R_{ij\times k}$ is negligible to leading order in the analysis). Similarly, equation (3.5) determining which equilibrium is stable along the polygynous edge continues to hold, where now $f_{PP\times P}=f_{MP\times P}=1$ given that only *P* males are present along this edge. Most importantly, polygynous-only colonies are always stable to the introduction of *MM* queens and *M* males, regardless of the values of $\alpha_{ij\times k}$, when the mating scheme is proportional to the product of the frequency of both mates.

**Figure 4.**
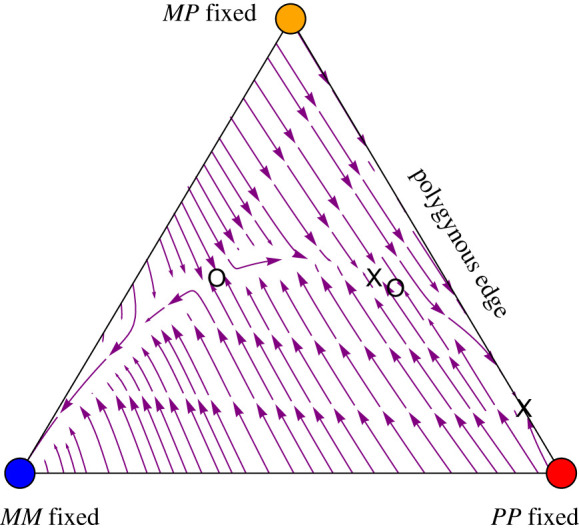
Stream plot showing the dynamics of *F. selysi* queens with sexual selection such that queens exhibit fixed-relative mating preferences [27]. Here, we illustrate a case when populations composed of only monogynous colonies or only polygynous colonies are both stable (condition (3.1) holds). The open circles mark unstable equilibrium points and the x's mark stable equilibrium points. Parameters: $f_{MM\times M}/f_{MM\times P}=96\;$ (an extremely strong preference), $f_{MP\times P}/f_{MP\times M}=f_{PP\times P}/f_{PP\times M}=1.47,\;V_{MM|ij\times k}^{\mathrm{female}}=0.46$, $V_{MP|ij\times k}^{\mathrm{female}}=0.69$, $V_{PP|ij\times k}^{\mathrm{female}}=0.33$, $V_{MM|MP\times M}^{\mathrm{female}}=V_{M|MP}^{\mathrm{male}}=0$ (complete maternal-effect killing) and $V_{a|ij}^{\mathrm{male}}=1$ (males have the same viability). (Online version in colour.)

In the cases discussed thus far (random mating, fertility differences and sexual selection with fixed-relative preferences), monogynous colonies can never invade when very rare. However, with very strong sexual preferences, *MM* queens can so strongly prefer *M* males that monogynous colonies can be maintained, once they rise to a sufficiently high frequency. In this case, a stable social polymorphism may exist under extreme preference differences (see electronic supplementary material for the specific description of parameter conditions required). Figure 4 illustrates one example, where *MM* queens prefer to mate with *M* males almost 100 times more than *P* males, allowing *M* to spread from intermediate frequency even though it cannot spread when rare. Importantly, a numerical search indicates that the degree of preference of queens for males of their own social form must differ between monogynous and polygynous queens in order to stabilize a social polymorphism. When monogynous and polygynous queens had the same degree of preference for males belonging to their own social form, a numerical search of one million random parameter sets found no stable social polymorphism. Note that with sexual selection and fixed-relative mating preferences [27], if *MM* queens have a strong preference for *M* males, then mating becomes effectively assortative, because *MM* females search until finding an *M* mate.

### (d) Assortative mating

Given the above results, we conjectured that a key feature necessary for both social haplotypes to spread when rare and maintain a social polymorphism is assortative mating, such that rare individuals can find and mate with similarly rare partners belonging to the same social form. A variety of mating schemes can produce assortative mating, for example when queens actively seek their preferred mate or when mating occurs near or within colonies.

We thus introduce assortative mating by social form, allowing mating among members of the same colony type. Specifically, we assume monogynous queens mate exclusively with *M* males at some frequency, $m_{M}$, and otherwise mate randomly, while polygynous queens mate with *P* males at some frequency, $m_{P}$, and otherwise mate randomly. We allow the two social morphs to mate assortatively at different rates because of empirical results indicating that monogynous queens have more often mated assortatively than polygynous queens [22]. With assortative mating by social form, we have:

3.6 $$\begin{array}{rl} R_{MM\times M} & =(1-m_{M})\;X_{MM}\;Y_{M}+m_{M}\;X_{MM},\\ R_{MM\times P} & =(1-m_{M})\;X_{MM}\;Y_{P},\\ R_{MP\times M} & =(1-m_{P})\;X_{MP}\;Y_{M},\\ R_{MP\times P} & =(1-m_{P})\;X_{MP}\;Y_{P}+m_{P}\;X_{MP},\\ R_{PP\times M} & =(1-m_{P})\;X_{PP}\;Y_{M}\\ \;\mathrm{and}\;R_{PP\times P} & =(1-m_{P})\;X_{PP}\;Y_{P}+m_{P}\;X_{PP}. \end{array}$$

In the absence of *MM* female or *M* males, we again have two possible equilibria on the polygynous edge, with only *P* males present and either *PP* queens or a *PP*/*MP* polymorphism, given by equation (3.2) (details of the stability analyses are given in the supplementary material). On this edge, the *MP*/*PP* equilibrium is stable if (3.3) holds, and otherwise the *P*-fixed equilibrium is stable.

With assortative mating, it is now possible, however, for the equilibria on the polygynous edge to be unstable to the introduction of *MM* queens and *M* males. Specifically, when haplotype *P* is fixed, monogynous colonies spread when rare if

3.7*a* $$V_{PP|PP\times P}^{\mathrm{female}}<\;m_{M}\;V_{MM|MM\times M}^{\mathrm{female}}.$$

Alternatively, if polygynous colonies consist of *PP* and *MP* queens at equilibrium (equation (3.2)), then monogynous colonies spread when rare if

3.7*b* $$\frac{V_{MP|MP\times P}^{\mathrm{female}}}{2}<m_{M}\;V_{MM|MM\times M}^{\mathrm{female}}.$$

Notice that the relative magnitudes of the left-hand sides of (3.7) determine which equilibrium is stable along the polygynous edge (equation (3.3)). Whichever value is larger determines the composition of polygynous colonies against which rare monogynous colonies must compete. In either case, the key feature is that rare *MM* queens are able to find and mate with rare *M* males, regardless of how rare they are. Only this feature allows monogynous colonies to escape the strongly absorbing polygynous edge caused by maternal-effect killing and to establish when rare.

In addition, under certain conditions, polygynous colonies can also spread within a fully monogynous population. Analysing the invasion of *P* into a population fixed on *M* involves solving a cubic equation for the leading eigenvalue (see electronic supplementary material). When there is complete assortment $\left(m_{M}=m_{P}=1\right)$, this cubic equation factors, allowing us to determine that *P* will spread if either of these conditions is met:

3.8*a* $$V_{PP|PP\times P}^{\mathrm{female}}>V_{MM|MM\times M}^{\mathrm{female}}$$

or

3.8*b* $$\frac{V_{MP|MP\times P}^{\mathrm{female}}}{2}>\;V_{MM|MM\times M}^{\mathrm{female}}.$$

These conditions for the spread of *P* when rare with complete assortative mating contradict the conditions for the spread of *M* when rare. Effectively, with complete assortative mating, the system quickly approaches two isolated sub-populations consisting of {*MM*,*M*} and either {*PP*,*P*} or {*PP*,*MP*,*P*} individuals (whichever polygynous set is more fit). Without genetic exchange between these sub-populations, either the monogynous sub-population {*MM*,*M*} is more fit and fixes or is less fit and disappears. Either way, a social polymorphism is not possible with complete assortative mating by social form. This was confirmed in a numerical search across one million randomly chosen parameter sets.

**Figure 5.**
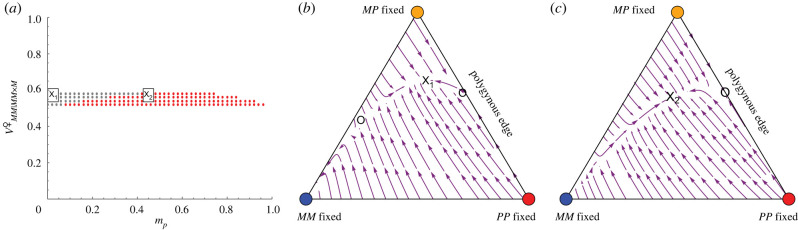
The maintenance of social polymorphism is possible with assortative mating by social form, here illustrated with complete assortative mating in monogynous queens and partial assortative mating in polygynous queens. Here, *MM* queens only mate with *M* males $\left(m_{M}=1\right)$. (*a*) The range of assortative mating in polygynous colonies $\left(m_{P}\right)$ and the viability of monogynous queens $\left(V_{MM|MM\times M}^{\mathrm{female}}\right)$ for which a stable social polymorphism persists when $V_{MP|ij\times k}^{\mathrm{female}}=1$ and $V_{PP|PP\times P}^{\mathrm{female}}=0.3$. Grey dots, both a social polymorphism and *M*-fixed equilibria are stable; red dots, only the social polymorphism is stable. (*b*) and (*c*) Stream plots for the parameter sets indicated in (*a*) ((*b*) at $x_{1}$, (*c*) at $x_{2}$). Open circles mark unstable equilibrium points, while $\times_{1}$ and $\times_{2}$ indicate stable internal equilibrium points. Other parameters: $V_{MM|MP\times M}^{\mathrm{female}}=V_{M|MP}^{\mathrm{male}}=0$ (maternal-effect killing) and otherwise $V_{a|ij}^{\mathrm{male}}=V_{ab|ij\times k}^{\mathrm{female}}=1$.

Thus, with either random mating $\left(m_{M}=m_{P}=0\right)$ or with complete assortative mating by social form $\left(m_{M}=m_{P}=1\right)$, a stable social polymorphism does not exist. With an intermediate rate of assortative mating by social form, however, a social polymorphism can result (figure 5). For example, if the *P*-fixed equilibrium is stable along the polygynous edge, it is possible for *M* to spread when rare if assortative mating is sufficiently high in monogynous colonies, as long as *MM* queens are fitter than *PP* queens, and for *P* to spread when rare if *MP* queens are fitter than *MM* queens. Both social haplotypes can invade when rare even if selection is weak (all $V_{a|ij}^{\mathrm{male}}$ and $V_{ab|ij\times k}^{\mathrm{female}}$ near 1, except for $V_{MM|MP\times M}^{\mathrm{female}}=V_{M|MP}^{\mathrm{male}}=0$ due to maternal-effect killing), as long as

3.9 $${\left(1-m_{P}\right)}^{2}<(1-m_{M})<\;V_{MM|MM\times M}^{\mathrm{female}}-V_{PP|PP\times P}^{\mathrm{female}}.$$

This result requires $V_{MM|MM\times M}^{\mathrm{female}}>V_{PP|PP\times P}^{\mathrm{female}}$ and monogynous queens mate assortatively at a very high rate (but not exclusively). Note that the more polygynous queens mate assortatively (the higher $m_{P}$, including $m_{P}=1$), the easier this condition is to satisfy.

#### (i) Complete assortative mating in monogynous queens

As queens heading mature monogynous colonies have exclusively mated with *M* males, while polygynous queens have mostly, but not exclusively, mated with *P* males [22], we first focus numerically on cases where $m_{M}=1$ and explore the conditions needed for the maintenance of a polymorphism. With complete assortative mating $\left(m_{M}=1\right)$ and when males are assumed equally fit, a stable social polymorphism is possible, but only when there is strong heterozygous advantage, as illustrated in figure 5. For some parameters, there are two separate stable equilibria (grey dots in figure 5*a*), one is a stable social polymorphism and the other is fixed for the *M* haplotype. These cases tend to be found when polygynous queens mate almost randomly (low $m_{P}$). Such systems are, however, prone to losing social polymorphism. For example, the colonization of new environments by monogynous queens could result in the loss of polygynous colonies, and such populations would then resist invasion by rare *P* haplotypes. When polygynous queens mate assortatively at a higher rate (intermediate $m_{P}$), the *P* haplotype is more likely to spread when rare, leading to cases where the only stable polymorphism involves a social polymorphism (red dots in figure 5*a*), which better resist loss of the polygynous type.

#### (ii) Incomplete assortative mating in monogynous queens

**Figure 6.**
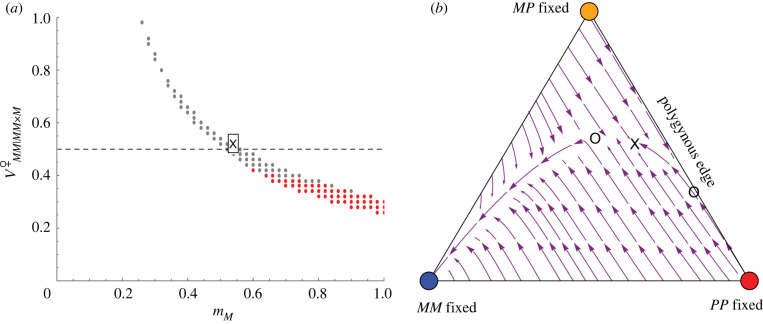
Partial assortative mating by both social forms allows for the maintenance of social polymorphism. (*a*) The range of assortative mating by social form in monogynous colonies $\left(m_{M}\right)$ and the viability of monogynous queens $\left(V_{MM|MM\times M}^{\mathrm{female}}\right)$ for which a social polymorphism persists. Above the dashed line corresponds to directional selection favouring the *M* haplotype in females, while below the dashed line, there is heterozygous advantage. Grey dots, both a social polymorphism and *M*-fixed equilibria are stable; red dots, only the social polymorphism is stable. (*b*) The stream plot for a parameter set (marked by an X in (*a*)) that permits a stable internal equilibrium denoted by an X in (*b*) $\left(m_{M}=0.54,\;V_{MM|MM\times M}^{\mathrm{female}}=0.52\right)$. Open circles mark unstable equilibrium points. Other parameters are as in figure 5 except $V_{MP|ij\times k}^{\mathrm{female}}=0.5,\;V_{PP|ij\times k}^{\mathrm{female}}=0.2$ and $m_{P}=0.5$.

Recent fieldwork has shown that monogynous queens sampled from mating swarms do mate with *P* males [23], with $m_{M}=\;∼0.65$, even though this cross is not observed in mature monogynous colonies [18,22]. We thus relax the assumption that $m_{M}=1$ and explore the conditions under which a polymorphism can be maintained. In the numerical cases explored, as the rate of assortative mating by monogynous queens declines, the *M* haplotype must be increasingly fit for a social polymoprhism to be stably maintained (recall that it is assortative mating that protects the *M* haplotype from being eliminated by maternal-effect killing in *MP* daughters produced when *MM* queens mate with *P* males). For example, with the parameters in figure 6*a*, there must be heterozygote advantage with *MM* queens less fit than *MP* queens under intermediate to high rates of assortment ($m_{M}$ above 0.6), but there must be directional selection favouring *MM* queens under low to intermediate rates of assortment ($m_{M}$ between 0.2 and 0.6), with no stable polymorphism possible for weaker assortment. Again, for each set of parameters that ensures a stable social polymorphism, the fixation of the *M* haplotype may (grey points in figure 6*a* and stream plot in figure 6*b*) or may not (red points in figure 6*a*) be a separate stable equilibrium (see electronic supplementary material).

In summary, we find that, under the right balance of parameters (as illustrated in figures 5 and 6), assortative mating of monogynous queens allows for the spread of monogynous colonies when rare. For example, if selection is weak (all $V_{a|ij}^{\mathrm{male}}$ and $V_{ab|ij\times k}^{\mathrm{female}}$ near 1, except for $V_{MM|MP\times M}^{\mathrm{female}}=V_{M|MP}^{\mathrm{male}}=0$ due to maternal-effect killing), for a social polymorphism to be maintained, this balance requires that both monogynous and polygynous queens mostly, but not always, mate assortatively, in such a way that equation (3.9) is satisfied. Although constitutive costs of assortative mating are incorporated in the viability terms, we have not incorporated frequency-dependent costs of assortative mating. If costs rise with the rarity of similar mates, we expect that it would be harder to maintain a social polymorphism because rare genotypes would pay that cost and be less likely to spread (particularly, rare monogynous types would be less able to spread near the polygynous edge).

The results above do not change substantially when assortative mating depends on genotype, rather than on social type, with *MP* queens mating equally with *M* and *P* males (except that the equivalent to condition (3.9) for a social polymorphism under weak selection is never satisfied; see electronic supplementary material). While assortative mating, by either social form or genotype, does allow the maintenance of a social polymorphism (figures 5 and 6), the conditions for stability are restrictive, requiring a delicate balance of selection among genotypes depending on the strength of assortment (e.g. figure 6*a*).

## 4. Discussion

Selfish genetic elements favouring their own transmission are ubiquitous across the tree of life, yet explaining their dynamics and observed frequencies in natural systems is challenging [6,31]. In short, when a genetic element drives, what prevents it from reaching fixation and becoming undetectable due to the lack of polymorphism? Maternal-effect post-segregational killers have been detected in ants [8], beetles [32], nematodes [33] and many bacteria, including endosymbionts [6,9]. In such systems, a maternally expressed toxin linked to a zygotically expressed antidote causes the death of progeny that did not inherit the element [9,34]. Simple population genetic models show that these selfish genetic elements are expected to increase in frequency and that high fitness costs of the elements are needed to prevent their fixation [31,35]. Supergenes controlling complex phenotypes are prone to harbour selfish genetic elements, yet the effect of drive on their evolutionary dynamics had not been modelled so far.

We have modelled the dynamics of a supergene controlling colony social organization in the Alpine silver ant, *F. selysi*, exploring a broad range of fitness functions and mating regimes and incorporating the transmission ratio distortion caused by maternal-effect killing by the *P* haplotype [8]. A key finding of the model is that maternal-effect killing creates such an imbalance in the selective forces acting that it is challenging to account for the long-term persistence of both social forms, i.e. monogynous colonies with a single queen and polygynous colonies with multiple queens. Indeed, there is no stable polymorphic equilibrium in any model with constant fitnesses and mating regimes that did not generate assortative mating. Essentially, maternal-effect killing prevents rare monogynous colonies from invading populations of polygynous colonies whenever rare *MM* queens mate predominantly with the common type of male (*P*), producing *MP* daughters that do not regenerate *MM* queens or *M* males.

For both social forms to be protected from loss when rare (i.e. for there to be no stable equilibrium with only one social form), we find that there must be strong assortative mating, so that *MM* queens frequently mate with *M* males even when both are rare, with the right balance of fitnesses involving strong selective differences among genotypes (e.g. figures 5 and 6) or very high, but not complete, assortative mating by both social types (equation (3.9)). Alternatively, there may be a stable social polymorphism alongside stable equilibria with only one social haplotype, which we observed with assortative mating (including with sexual selection when fixed-relative preferences were so strong that assortment arose). The latter cases are, however, more prone to losing social polymorphism, if by chance one social form goes locally extinct. Whether the restrictive conditions allowing a social polymorphism are satisfied in Alpine silver ants remains an open question, as discussed below.

In many supergenes, one haplotype is a homozgyous lethal, which selects for disassortative mating [36]. The Alpine silver ant system is unusual, as both homozygotes are viable. Moreover, maternal-effect killing by the *P* haplotype may select for assortative mating by *MM* queens for *M* males and *MP* queens for *P* males, respectively. In line with that prediction, effective mating in Alpine silver ants appears to be strongly assortative: when analysing queens heading mature field colonies, all *MM* queens had mated with *M* males, while *MP* and *PP* queens had mated mostly with *P* males (77%; [18,22]). The proximate mechanisms underlying this pattern of assortative mating remain unclear. In mate choice experiments in captivity, queens and males mated randomly with respect to social form [24], but the experimental conditions may have interfered with natural mate choice. In the field, queens and males from each social form fly away from their natal colony to join mating swarms, where *MM* queens mate mostly assortatively ($m_{M}=\;∼0.65$, based on mating swarm data in [23]). Queens in monogynous colonies do not mate with members of the same colony [22]. Hence, the assortative mating pattern observed in the field is consistent with a major condition for stability in the model, but we do not know whether and how *MM* queens actually seek out and mate preferentially with *M* males when both are rare, as required to maintain the social polymorphism. Assortative mating appears easier to achieve within the polygynous social form, as *PP* or *MP* queens as well as *P* males may mate within or close to their natal colony.

Regarding the strength and direction of selection, it is extremely difficult to obtain precise estimates of fitness, given that Alpine silver ant queens are long-lived and iteroparous [16]. Moreover, the two social forms differ in morphology, sex allocation, colony size and other life-history traits, which hampers fitness comparisons [16,37,38]. Queens from alternative social forms may well differ in fitness, as they typically use alternative modes of dispersal and colony founding (independent colony founding versus budding; [28]). Queens from monogynous colonies disperse on the wing and found colonies independently, while queens from polygynous colonies have the additional options of staying in their natal colony, seeking adoption in other colonies, founding colonies with the help of workers or in assocation with other queens [39]. Queens from monogynous colonies are more fertile and more successful at independent colony founding in harsh ecological conditions than queens of polygynous origin [24,29,40].

The accumulation of deleterious mutations in the non-recombining supergene can cause fitness differences among genotypes, generating associative overdominance that can help stabilize polymorphisms [11]. As noted by Berdan *et al*. [11], the accumulation of deleterious mutations is a double-edged sword, capable of stabilizing polymorphisms, but only during time periods that selection remains balanced across the haplotypes. In Alpine silver ants, the contribution of deleterious mutations is likely to be lessened by expression and efficient purifying selection in haploid males. Deleterious mutations that primarily affect females could, however, accumulate in the *P* haplotype yet be masked in *MP* polygynous queens; by contrast, the accumulation of deleterious mutations in the *M* haplotype always lowers the fitness of monogynous colonies, as there is never masking in *MM* queens. Recent studies in the field and laboratory revealed that *PP* females have lower survival, fertility and fecundity than *MP* females (P Blacher, O De Gasperin, G Grasso, S Sarton-Lohéac, R Allemann and M Chapuisat 2022, unpublished results). Overall, life-history and dispersal differences between social forms, combined with a recessive genetic load that can accumulate in the *P* haplotype, might thus create fitness differences between supergene genotypes that contribute to stabilizing the polymorphism (e.g. as seen in figure 5).

While general in many respects, our model does not investigate all forms of frequency- or density-dependent selection. Frequency-dependent selection may arise from antagonistic interactions [41]. For example, if one social form is more aggressive than the other, it may be favoured when rare and disfavoured when common. Spatially varying selection due to habitat heterogeneity may also stabilize the polymorphism [20,42]. In particular, monogynous queens may be better at dispersing and colonizing novel or patchy habitats, while polygynous queens may outperform monogynous ones in old, continuous and saturated habitats, due to their larger colony size and possibility of establishing novel colonies by budding [16,20,28]. Such ecological niche differentiation could maintain both types, just as it can maintain different species, but it would not explain why mating between social forms persists, as the continued gene flow between the types prevents further genetic adaptation of each social form to its specialized ecological niche.

Supergenes, such as found in Alpine silver ants, typically affect multiple morphological, physiological and behavioural traits [1], and over time they tend to accumulate recessive deleterious mutations [5] and selfish genetic elements [8]. The *P* haplotype of *F. selysi*, which is derived and differs by several inversion from the ancestral *M* haplotype, causes selfish post-segregational killing [8,19]. The supergene that controls social organization in the fire ant *Solenopsis invicta* also causes unusual patterns of segregation distortion and genotype-specific mortality [7,43,44]. In mice, the *t*-haplotype not only distorts segregation, it also affects sperm competition and increases dispersal propensity [26,45,46]. The *Segregation Distorter* supergene in fruit flies has diverse effects on fitness and sex ratio [47]. In many empirical cases, the driven or positively selected supergene haplotype is a homozygous lethal but has lower frequencies than expected by simple models [48]. Although the details of each system differ, our model shows that explaining the long-term maintenance of selfish supergenes is not trivial. Overall, the drive induced by maternal-effect killing in Alpine silver ants destabilizes the polymorphism and prevents rare monogynous colonies from invading populations fixed for polygynous colonies. Under the broad range of models considered here, a stable polymorphic equilibrium can only be reached under a regime of strong but not complete assortative mating, combined with the right balance of fitnesses for alternative supergene genotypes.

## Data Availability

The data are provided in electronic supplementary material [49].
